# Smokers’ knowledge and perception of electronic cigarettes (e-cigarettes): a qualitative study of non-quitting smokers in a North London general practice

**DOI:** 10.1017/S1463423618000439

**Published:** 2018-07-02

**Authors:** Vanessa Vasconcelos, Hazel Gilbert

**Affiliations:** 1 Medical Student, Department of Primary Care and Population Health, UCL Medical School, London, UK; 2 Principal Research Fellow, Department of Primary Care and Population Health, UCL Medical School, London, UK

**Keywords:** e-cigarettes, ENDS, smokers’ knowledge, smokers’ perception

## Abstract

**Background:**

The introduction of electronic cigarettes (e-cigarettes) has provided smokers with an alternative source of nicotine. Interest and use of the device has increased exponentially in the last decade with an estimated 2.9 m adult users in Great Britain. Research so far on the attitudes and perceptions of smokers to this new product has largely focussed on the views of current e-cigarette users, smokers attempting to quit and former cigarette smokers.

**Aim:**

This study aimed to explore the views of current tobacco smokers who were not using e-cigarettes and not looking for a cessation method, their understanding and knowledge of e-cigarettes, and their views of e-cigarettes as a smoking cessation aid provided by the National Health Service (NHS).

**Methods:**

Semi-structured in-depth interviews were conducted with 14 patients from a general practice in North London, who smoked conventional tobacco cigarettes on a daily or weekly basis, over 18 years old. An iterative approach allowed for constant data analysis using a thematic approach throughout the data collection stage, and generated four recurring themes.

**Findings:**

E-cigarettes were primarily seen as a smoking cessation device, with the supply of nicotine viewed as a benefit helping to reduce withdrawal symptoms, although for some participants this supply could also be a hindrance to dealing with their addiction. Despite uncertainty about the components, e-cigarettes were mostly viewed as healthier due to their lack of carcinogens, tar and smoke inhalation. The lack of reliable information and strong evidence for both the effectiveness and the safety of e-cigarettes led participants to be apprehensive about their provision by the NHS, and acted as a barrier to their use as an aid to quitting. The recurring appeal for more information regarding e-cigarettes make it clear that further high-quality research is urgently needed in this field to provide reliable and accurate information to smokers.

## Introduction

In 2015, 17.8% of individuals in the United Kingdom over the age of 16 reported being smokers (Office for National Statistics, 2017) and an estimated 16% of deaths over the age of 35 years were as a result of smoking [National Health Service (NHS), 2017]. The importance to the NHS of encouraging smokers to quit is illustrated by the 1.8 million prescriptions for smoking cessation medications dispensed in England in 2015/2016 (NHS, 2017).

Nicotine is the substance in tobacco that causes smokers to become dependent. ‘People smoke for nicotine but they die from the tar’ (Russell, 1976), and this is the basis for the development of nicotine replacement therapy (NRT) as an aid to quitting. Originally introduced as nicotine gum, NRT is now available in many forms, including patches, inhalers and nasal sprays, and can help smokers by reducing the craving for nicotine while allowing them to deal with the behavioural and habitual aspects of smoking. The Stop Smoking Services (SSS) in England provide free help and advice to smokers wishing to quit, combining behavioural counselling, in either one-to-one or group settings, with NRTs and other stop smoking medications (National Institute for Health and Care Excellence, 2013).

The introduction in the last decade of electronic cigarettes or ‘e-cigarettes’ has provided an alternative source of nicotine. These e-cigarettes, also known as electronic nicotine delivery systems, are devices which use battery power to provide vaporised nicotine which is inhaled by the user. They take a range of forms, with varying designs, nicotine concentrations and vapour release methods. Use of these commercial products has increased exponentially in the last decade, and their increased popularity with smokers may be accounted for by their similarity to conventional tobacco cigarettes. Surveys have demonstrated an increased awareness and use of e-cigarettes within the European Union and United States (Filippidis *et al*., 2017; Huerta *et al*., 2017), with an estimated 2.9 m adult users in Great Britain (Action on Smoking and Health, 2017).

Since their introduction and rapid increase in use, e-cigarettes have been the topic of much debate in both academic and public health communities. The World Health Organisation has outlined concerns regarding their use, and these include the health risks and safety for users, their effectiveness as a smoking cessation aid, concerns about their ability to initiate nicotine use in never smokers, particularly in younger users, and also the potential for continued nicotine addiction in smokers [World Health Organisation (WHO), 2016]. While e-cigarettes are generally regarded as less harmful than tobacco, a systematic review concluded that due to methodological problems, lack of long-term follow-up and conflict of interest, no distinct conclusions can be made and more research on the safety of e-cigarettes is urgently needed (Pisinger and Dossing, 2014).

Smoking cessation is frequently cited as a reason for e-cigarette use (Dawkins *et al*., 2013), but despite considerable research, evidence regarding effectiveness of e-cigarettes to aid smoking cessation remains inconclusive due to the low quality of the studies (Gualano *et al*., 2015; Hartmann-Boyce *et al*., 2016; Khoudigian *et al*., 2016; Malas *et al*., 2016). Evidence for their effectiveness is also inconsistent and contradictory amongst different populations (Hirano *et al*., 2017; Zawertailo *et al*., 2017). Although overall the literature suggests e-cigarettes may be helpful for some smokers for quitting or reducing smoking, studies demonstrating a positive relationship between e-cigarette use and smoking cessation are short term (Dawkins *et al*., 2012), have insufficient statistical power (Bullen *et al*., 2013), or are cross-sectional or observational studies (Brown *et al*., 2014). There are few robust randomised controlled trials and more carefully designed and scientifically sound studies are needed (Malas *et al*., 2016).

Besides the need for high-quality research into the health consequences and the effectiveness of e-cigarettes as a smoking cessation aid, it is necessary to understand the perceptions of the public towards e-cigarettes with respect to both the health implications and safety and their use as a cessation aid. Surveys exploring the views of current e-cigarette users highlight their belief that e-cigarettes are healthier, improve their breathing and are safer in comparison to tobacco (Dawkins *et al*., 2013; Wong *et al*., 2016). These beliefs were particularly prevalent amongst younger, more educated smokers and current or former smokers (Li *et al*., 2013; Tan and Bigman, 2014; Pepper *et al*., 2015; Rutten *et al*., 2015). However, these positive views regarding the reduced harm in e-cigarette use were less often expressed by former and never smokers of e-cigarettes (Tan *et al*., 2016). The regulatory environment has also been found to affect the perception of harm from e-cigarettes (Yong *et al*., 2017). The importance of the behavioural resemblance to conventional cigarette smoking and the fewer side effects in comparison to other smoking cessation methods were viewed as benefits of e-cigarettes amongst current tobacco smokers (Simmons *et al*., 2016). Nevertheless, concerns regarding the safety and efficacy of e-cigarettes were raised by smokers seeking cessation support, and were more evident in never users of the product (Sherratt *et al*., 2016). In addition, varied views have been expressed among smokers regarding the risk of persistent nicotine addiction whilst using e-cigarettes (Rooke *et al*., 2016). The evidence is conflicting, leading Tomashefski (2016) to conclude that high-quality evidence is limited and further research may help to clarify the perception of the effectiveness and safety of e-cigarettes.

An increase in e-cigarette use amongst patients has been observed by physicians and healthcare professionals, with some success with smoking cessation (Hiscock *et al*., 2014; Kandra *et al*., 2014). However, despite considering e-cigarettes safer than tobacco, health professionals have also expressed concerns about safety and are unwilling to recommend the use of e-cigarettes to patients (Gorzkowski *et al*., 2016; Marques Gomes *et al*., 2016; Van Gucht and Baeyens, 2016). Currently the SSS does not provide e-cigarettes as part of the service.

Research so far has largely focussed on the attitudes and perceptions of current e-cigarette users, smokers attempting to quit and former cigarette smokers. However, there is limited qualitative research exploring the perception of e-cigarettes in current smokers who are not seeking cessation methods. The aims of this study were to qualitatively explore: (1) current tobacco smokers’ understanding and knowledge of e-cigarettes; (2) the factors that might influence or discourage their use in this group; (3) current tobacco users’ views of e-cigarettes as smoking cessation devices; and (4) their views on e-cigarettes as products provided by the NHS as a smoking cessation aid. By using a qualitative rather than a numerical approach we aimed to extract deeper insight into the ideas and views of current smokers not using e-cigarettes and not immediately looking for a cessation method.

## Methods

### Design and setting

We conducted a qualitative analysis of semi-structured in-depth interviews (Riemer *et al*., 2012) with patients from a general practice in North London. This practice ‘flagged’ patients as smokers on their medical system and employed a nurse specialising in smoking cessation. Interviews took place during March 2015. The study was approved by the NRES Committee Yorkshire & The Humber – Bradford Leeds REC.

### Participants and recruitment

Participants were current smokers, defined as those who smoked conventional tobacco cigarettes on a daily or weekly basis, over 18 years old. Ex-smokers, those who did not speak English, and current users of e-cigarettes were excluded.

Participants were opportunistically recruited using two methods. First, as smokers attended appointments at the practice they were informed of the study and provided an envelope containing participant information sheet and consent form by the GP/nurse. Second, posters were placed in the waiting room directing the patients to pick up the envelope with the same information from the reception or their nurse/GP. Both methods allowed participants to register their interest through a reply slip or contact the researcher directly. A week was given between the patient receiving the information and the researcher contacting them, to answer any questions and arrange an interview time and location, allowing the patient time to read the information and to contact the researcher themselves. To reach the target sample size of 10–15 participants allowing for ‘theoretical saturation’ (Charmaz, 2006), ~50 packs of information were created and handed out at the practice. Recruitment was terminated after 14 interviews as theoretical saturation had been reached.

### Procedure

Interviews took place in an available room at the practice. This ensured familiarity of the setting for participants. To encourage participation, the choice of a convenient venue outside the GP practice was also offered to patients. Consent was obtained for participation and audio-recording of the interview, and participants were informed of complete anonymity.

The interview schedule comprised a range of open-ended questions to address smokers’ general understanding of e-cigarettes, their perception of their use as a smoking cessation device and their opinions on their inclusion into SSS. Questions were designed to stimulate discussion and where necessary, prompts were used to encourage detailed responses. A pilot interview was conducted to verify the choices of questions and to determine the quality of the audio-recording equipment before proceeding with the interviews.

Due to the iterative nature of the study, minor modifications were made to the questions to tailor for emerging ideas throughout the interviews, whilst continuing to achieve the aims. The use of a vignette was employed to provide a fictional situation that the participant could comment on and was used as a method to stimulate the discussion. All interviews were audio-taped and carried out by one researcher (V.V.), who also made field notes of participants’ behaviours and any emerging themes to highlight in future interviews. The researcher had no previous affiliations to e-cigarettes, nor were they a user of the devices, or a smoker.

### Data analysis

The interviews were transcribed verbatim, and the final data consisted of the transcribed interviews and the researcher field notes, which included any non-verbal communications and potential theme ideas that were emerging throughout the interview. Data analysis was conducted by the first author and discussed and reviewed by the second author to improve reliability and reduce bias. The Thematic Framework Approach (Pope *et al*., 2000) was chosen to analyse the data as the most suitable method for applied research with explicit objectives, and appropriate for time limited research and NVIVO software used. This method involved multiple steps: (1) familiarisation with the data by re-reading transcripts; (2) identifying initial themes within all transcripts; (3) indexing the data for the themes identified in step two; (4) charting the data which involved reorganising the data and collecting them into relevant themes and groups; and (5) mapping and interpreting the themes, that is combining and re-arranging the identified themes to form a hierarchy of themes and subthemes. Throughout this process the most important themes were identified based on the aims of the study, which led to the final four themes and subthemes. The iterative approach allowed for constant data analysis throughout the data collection stage, highlighting themes that could be explored in future interviews known as theoretical sampling (Charmaz, 2006). It was intended that by the end of the interviews no more new themes emerged, providing theoretical saturation.

## Results

All interviews were carried out in a room within the practice, with no participant choosing a different location, and the interviews lasted between 22 and 57 min. Eight of the 14 participants were male; the age range was 20–80 years (mean=54.2). Although eight had used e-cigarettes in the past, none were current users. Five said they planned to quit within the next 30 days, but had not set a quit date, nor commenced any form of plan. In all, 13 participants recognised e-cigarettes, however, one participant had very little understanding and had never been exposed to one (Table 1).

**Table 1:** Participant characteristics

Participant	Age	Sex	Smoking frequency	Number of cigarettes a day (when smoke)	Planning to quit in next 30 days	Previous use of e-cig
1	56	Male	Daily	5	Yes	Yes
2	72	Male	Daily	30	No	Yes
3	72	Female	Daily	20	Yes	Yes
4	34	Female	Daily	7	No	
5	74	Male	Daily	30	Yes	
6	47	Male	Daily	10	No	Yes
7	80	Female	Daily	3	Yes	
8	56	Male	Daily	25	No	Yes
9	47	Female	Daily	20	No	
10	72	Male	Daily	20	No	
11	20	Male	Occasionally (weekends)	10	No	Yes
12	59	Female	Daily	10	No	Yes
13	35	Female	Daily	20	No	
14	35	Male	Daily	5	Yes	Yes

e-cig=electronic cigarettes.

Data analysis of the transcribed interviews and field notes generated four themes and six subthemes (Table 2). The quotes within the text are taken verbatim from the interviews.

**Table 2:** Results of the thematic framework analysis presented as themes and subthemes

	Themes	Subthemes
1	Components of e-cigarettes	
2	Health perspective of e-cigarettes	
3	Using e-cigarettes as a smoking cessation method	(a) Dealing with the addiction (b) Effectiveness of e-cigarettes to quit smoking (c) Encouraging the uptake of smoking
4	The role of the NHS and scientific research	(a) Sources of information (b) Lack of evidence (c) Role of the NHS

e-cigarettes=electronic cigarette; NHS=National Health Service.

## Theme 1: components of e-cigarettes

A variety of views and interpretations emerged regarding the components of e-cigarettes. While many participants understood that e-cigarettes did not have the same harmful substances as ordinary cigarettes such as tar and carcinogens, several were cautious of additional components within an e-cigarette which may not necessarily be present in an ordinary cigarette.

‘*I know it [e-cigarette] is supposed to not have the carcinogenic and things like that, and it is basically nicotine and fancy tastes if you want strawberry you get strawberry*’.

(Participant 11005)

‘*I read some research that says they have some harmful waxes in them like lethal to humans so I don’t know if that is true or not, and also they have got nicotine in them, but smaller doses maybe*’.

(Participant 25011)

The majority were also aware that e-cigarettes, like ordinary cigarettes, contained nicotine. However, in some cases, participants were not sure whether what they termed ‘vapour cigarettes’ contained nicotine at all, suggesting a misunderstanding that may be common in the smoking population.

‘*I think this is why they brought the vapour cigarette, because I mean, the vapour cigarettes have no nicotine at all, it is just the flavour, I mean you can get hundreds of different flavours for them*’.

(Participant 03001)

‘*It might be water vapour it might be laced with nicotine but we don’t know what else is in there [electronic cigarette]*’.

(Participant 31014)

In general participants’ knowledge of e-cigarette content was sparse, and uncertainty to the truth was evident. A number expressed their lack of knowledge and confusion leading to suspicion towards their use.

## Theme 2: health perspective

Despite the uncertainty about their components, e-cigarettes were regularly viewed as healthier due to their lack of carcinogens, tar and smoke inhalation. Nicotine was also considered by a few participants, who understood the health implications of nicotine, such as circulatory problems, but these were less concerning than the health implications of the tar and carcinogens.

‘*I would imagine they are [healthier] because I can’t see how they have the same volume of pollutants you know. And there is probably no tar and things like that which is obviously massively bad*’.

(Participant 31014)

‘*look at a cigarette with the filter, look at the cigarette after you have smoked it with a filter on, you can see what is happening there, you know look at my teeth, you know you can see what is happening there, you don’t get that with the vapour[electronic cigarette]*’.

(Participant 12006)

‘*And nicotine is obviously not good for the circulation or anything else, but it doesn’t have the other carcinogens that tobacco does have (…) the nicotine is not healthy, so they are never going to be healthy, they [electronic cigarettes] are not as unhealthy as the straight tobacco*’.

(Participant 12006)

E-cigarette use as a method of smoking cessation was often a contributing factor to the positive health views. As one participant described, ‘*I would have thought the fact that they made the person give up smoking would outweigh anything that probably that possibly could come up*’ (Participant 03002). Lack of knowledge remained an issue and caused some participants to question the health benefits of e-cigarettes, feeling that a healthier option did not exist, and describing them to be as bad as ordinary cigarettes.

‘*I sat there and I thought, now you are inhaling that oil so surely that oils going to clog your lungs as much as the cigarette so I stopped using it*’.

(Participant 09003)

‘*I don’t know what they contain, or… what the outcome is, are they healthier? I don’t know how they work to be quite honest. Is there tobacco in them or, I really don’t know*’.

(Participant 17007)

‘*I don’t know if they are worse than cigarettes, are they causing more harm than normal cigarettes, even though normal cigarettes cause you harm, are these [e-cigarettes] causing you more harm, that is why I am still smoking normal cigarettes*’.

(Participant 17008)

It was recognised that the health implications were not restricted to the users of e-cigarettes but may also affect those surrounding them. On this issue, many participants considered e-cigarettes to be harmless, and as a result it would have been anticipated that participants would accept the use of e-cigarettes in locations where tobacco cigarette smoking is banned. However, this was not the case, with many participants opting for the use of e-cigarettes in the same locations as ordinary smoking.

‘*I don’t think they will harm anybody around about you*’.

(Participant 03001)

‘*if I am walking behind somebody smoking cigarette even as a smoker I hate that and think it is disgusting because I have second hand smoke going in my face, at least with an e-cigarette I won’t have that*’.

(Participant 31014)

‘*I wouldn’t use it in the bus or restaurant, yeh,[use it] like you do now with normal smoking, you know, I would apply the same with it, I wouldn’t do all that (…) well people think you are still smoking normal cigarettes and people will; get offended so I just wouldn’t*’.

(Participant 17008)

## Theme 3: using e-cigarettes as a smoking cessation method

All participants recognised that e-cigarettes had a primary use for helping to quit conventional cigarettes, although different factors influenced their perception of their use as alternatives, highlighted in the following subthemes.

### Dealing with the addiction

E-cigarettes were discussed throughout as a tool to attempt to deal with their addiction to tobacco. The majority of participants understood that nicotine formed the basis of their addiction and triggered their craving. While a few believed the supply of nicotine to be a benefit of e-cigarettes as it helped to reduce withdrawal symptoms whilst attempting to quit smoking tobacco, others raised concerns that using this product was replacing one addiction with another, and not dealing with the true problem of addiction to nicotine. Participants were worried that their use would cause greater addiction as users would be unaware of how often and for how long they would be drawing on the e-cigarette. This same axiom was applied when discussing the locations where e-cigarettes are smoked and the effects of being able to smoke them in numerous places and amongst people, leading to fewer restrictions on their nicotine habit.

‘*because you, it is not quite like, giving, the other[option] is you just cut yourself off from it [cigarettes] completely, so you get the withdrawals… but you get much less of that when you do it with the electric one*’.

(Participant 03002)

‘*I wouldn’t get that, rid of that nicotine need and addiction, because there is nicotine in it and so I will get addicted to that [e-cigarettes] instead of cigarettes*’.

(Participant 31013)

‘*I would love to say oh well, you know I want a cigarette, so I will have an e-cig or a vapour [instead]. I have got to stop altogether [e-cig and cigarettes]*’.

(Participant 03001)

‘*I am assuming that at the end of having a cigarette I have had enough, I don’t want to light another one straight away, but with the e-cigarettes there isn’t that stopping point, there isn’t the ‘dosage’ as it were (…) I would have a worse addiction to nicotine (…) I have said they are great these e-cigarettes but I just feel I could get a bit actually quite addicted to nicotine more so than I am now*’.

(Participant 12006)

‘*I can sit around my folks, my dad has had TB all sorts, he survives on 1/3 of one lung, but I can sit there at the dinner table with him and use an e-cigarette and he doesn’t mind, he doesn’t feel it whereas he feels cigarettes, so… that’s my concern is that I would smoke more, it would actually, because of the ability to use it in more places I would smoke more*’.

(Participant 12006)

### Effectiveness of e-cigarettes to quit smoking

Although the use of e-cigarettes to reduce withdrawal symptoms was seen as helpful, other aspects of their effectiveness as a smoking cessation aid were more equivocal. Their resemblance to ordinary cigarettes, and the ‘hand to mouth’ action, an uncommon feature amongst prescribed NRTs, was viewed overall as a benefit by many participants. Consequently, e-cigarettes were seen as more effective than current NRTs cessation and this would influence the choice of therapy. There was however, a concern that relapse may occur as a result of such resemblance while other participants did not agree that e-cigarettes resemble cigarettes due to their various flavours.

‘*cos see my problem is I have to have something to do with my hands, so I have tried the lozenges and the gum things before but it doesn’t take that, you need this bit [smoking gestures]*’.

(Participant 09004)

‘*For people who are trying to quit, if you are offering nicotine it’s the electronic ones that are going to work because they feel like you are still smoking whereas all the others don’t, they don’t deliver nicotine at that rate to the brain. So personally I think I would have a lot more success with an e-cigarette than any of this other stuff*’.

(Participant 12006)

‘*The patches don’t seem to work (…) they may take the nicotine craving away, but they don’t take the craving away for using your hands [simulates smoking gestures]. This is why I got one of these e-cigarettes [past use]*’.

(Participant 03001)

Another influencing factor was the cost of e-cigarettes ‘they are expensive, so to try and do trial and error it is sort of like I could buy two packets of fags for what that is costing me’ (Participant 18009). Some participants were aware of the many companies and different types of e-cigarettes that were available, but there was confusion as to which would suit them best, and be more effective, leading to a reluctance to experiment.

While many of the participants believed e-cigarettes would positively influence the cessation of smoking, there was no universal agreement on this point. Several discussed their past use of e-cigarettes, and this, alongside knowledge of other people’s experience of e-cigarettes, caused some to question the effectiveness of e-cigarettes.

‘*I think if you smoke an e-cigarette you are more likely to go back to smoking ordinary cigarettes (…) if you lost it you are more likely to go and buy 10 cigarettes from the garage (…) I don’t think it would be easier to quit, they are easier to use, because they give you the sensation of smoking, which I think will make you more liable to go back to smoking, than quitting all together*’.

(Participant 03001)

‘*I don’t think they stop the craving for a cigarette (…) I have known a lot of people who have bought them and they are still smoking*’.

(Participant 09003)

‘*I would still be addicted, I would still do that, I don’t think I would quit completely*’.

(Participant 31013)

### Encouraging the uptake of smoking

A few participants described the effect of e-cigarettes not only on smokers but to people who had no previous smoking habits. They believed that e-cigarettes were influencing the wrong audience and consequently could lead to an unprecedented addiction in those with no previous smoking habits or in the younger population, including children, particularly due to the use of flavourings in e-cigarettes.

‘*I have friends which they never smoked and like one girl for example had a boyfriend she wanted him to stop, then she made him go on the e-cigarettes and now she is walking around with an e-cigarette, inhaling that which she never smoked. And she was against cigarettes*’.

(Participant 31013)

‘*Where it might be a bad thing is are they due to their flavours and the number of flavours and the sweetness in those flavours are they encouraging people who are younger like teenagers and such to smoke or vape where they wouldn’t have necessarily smoked previously. So that is a risk*’.

(Participant 31014)

## Theme 4: the role of the NHS and scientific research

The emphasis when discussing the role of the NHS was the lack of reliable information and evidence.

### Sources of information

Many of the participants knew where they could purchase e-cigarettes and that they were widely available in local shops, supermarkets and shops designed primarily for the sale of e-cigarettes. As one participant put it ‘*you aren’t far from an e-cigarette*’ (Participant 31014).

Many were more likely to visit their GP or a pharmacist to obtain advice and a prescription, although this was largely if they were considering quitting using prescribed NRT. As a result, primary sources of information on e-cigarettes were either the chemist, the internet, such as forums, or e-cigarette shops, despite scepticism and a lack of trust regarding the latter two. While participants were clear that reliable information should be sought from GPs, health professionals were not seen as particularly knowledgeable about e-cigarettes and considered uninformed and naïve. There was, in general, a lack of confidence about where to get reliable information. Regarding the contents of e-cigarettes they would have to rely on the packaging for information.

‘*To his doctor…not the man behind the counter in the shop because he just wants to sell you it, he ain’t going to tell you this is harmful this is that, he wants to get your money*’.

(Participant 09003)

‘*not a lot of [doctors know about these], no, because I don’t think they have looked into them enough*’.

(Participant 09003)

‘*I think the pharmacist can tell you a lot about the patches and the Nicorette stuff but I don’t think they can tell you much about the e-cigarettes*’.

(Participant 03001)

### Lack of evidence

A particular issue was the lack of evidence and research to support their effectiveness as a smoking cessation aid, their safety and their components. This concern was expressed by many participants and played a leading part in their apprehensiveness towards e-cigarettes.

‘*they were just saying they don’t know and they haven’t been tested, the effects are of using them, there is a lot of reports people have given up using them everywhere you look, because nothing on the government or the NHS saying that this product is good for you to give up as many people have given up, there is not a solid body behind it*’.

(Participant 17008)

A few compared the introduction of e-cigarettes to the introduction of conventional manufactured cigarettes, and their popularity and promotion as healthy at that time. As a result they were aware of the lack of research into the long-term effect of e-cigarettes and highlighted the need for such research.

‘*like cigarettes used to be advertised and everybody used to smoke and manufacturers pushed for people to buy and smoke. And later it came out lots of disease and stuff, now with e-cigarettes everybody pushing for e-cigarettes advertisement and manufacture who is making them and I am worried this is only for making money of people*’.

(Participant 31013)

‘*we don’t know how that still affects the body after 20 years, you know there isn’t 20 years worth of research for example*’.

(Participant 31014)

### Role of the NHS

Some participants agreed that e-cigarettes could be utilised by the NHS prescription services, particularly as many qualified for free prescriptions and would therefore benefit. However, the lack of confidence and research support for both the effectiveness and the health perspective of e-cigarettes influenced many participants to be apprehensive about their provision by the NHS. Only if these issues were cleared would they be considered acceptable. Whilst discussing participant’s preference of e-cigarette it also emerged that they had no guidance with which to choose. For example, one participant, when discussing previous use of e-cigarettes, described the uncertainty of choosing the nicotine strength of an e-cigarette, and hoped that the NHS or a governing body would provide such information.

‘*if they are licenced and if there is an agreed “this is what goes in them” and the people manufacturing them actually have to abide by that then fair enough, then they certainly have a place, because they are basically the same as that [points at inhalator] in a way*’.

(Participant 09004)

‘*you know I didn’t pick the top one, I just picked the second one from the top, but I didn’t know, because there is nothing there is there to tell you, no there is no guide, that is what I was saying from the beginning, there is no guidance from the government or the NHS about them*’.

(Participant 17008)

## Discussion

In this study we explored the perception of e-cigarettes in patients who were current tobacco smokers at a North London General Practice. An overarching theme to emerge from these interviews was a general lack of knowledge about e-cigarettes and uncertainty about their content and safety. It was this lack of knowledge that led to diverging views and statements.

A general understanding of the role of nicotine as the cause of their addiction to tobacco, previously reported among current e-cigarette users (Etter and Bullen, 2011; Dawkins *et al*., 2012; Dawkins *et al*., 2013) was recognised by most participants in this sample. In this respect, e-cigarettes were primarily seen as a smoking cessation device, due to their nicotine content. Although considered a benefit, this nicotine supply was also viewed as a disadvantage, not helpful in terms of breaking the addiction to nicotine, and possibly leading to dependence on e-cigarettes or subsequent relapse to tobacco. In addition, participants were concerned about the resemblance of e-cigarettes to conventional cigarettes in the ‘hand to mouth’ action which does not deal with the habitual aspect of smoking, again supporting previous findings (Rooke *et al*., 2016).

The health aspects of e-cigarettes were a salient issue. They were generally considered healthier options in comparison with tobacco, notably due to perceived lack of carcinogens and tar, supporting previous research highlighting the same belief in e-cigarette users (Dawkins *et al*., 2013; Goniewicz *et al*., 2013; Tomashefski, 2016) and also in current tobacco smokers (Sherratt *et al*., 2016). However, doubts were expressed due to the lack of knowledge of their make-up, and in some cases e-cigarettes were viewed equally as harmful as conventional cigarettes. Participants were also uneasy about the unknown health implications of long-term use. These findings also emphasise that the concerns raised by quitters, in particular, quitters who were non-users of e-cigarettes (Sherratt *et al*., 2016), were also extremely prevalent within this sample of current tobacco smokers adding strength to previous reports that uncertainty, ambiguity and flawed beliefs about e-cigarettes are widespread in smokers (Rooke *et al*., 2016; Sherratt *et al*., 2016).

An interesting finding from these data was that a number of these current smokers were also aware of and concerned about the risks involved in passive smoking, and appreciated the lessening of the effects of smoking to the surrounding public. However, this did not lead to the acceptance of using e-cigarettes in locations where tobacco smoking is not permitted and there were some conflicting views regarding where their use should be allowed, reflecting perhaps that the stigma and social unacceptability of smoking (Stuber *et al*., 2008; Graham, 2012) would still apply to e-cigarettes. There was also a reassuring recognition of other issues, which perhaps followed on from their understanding of addiction. The risk of influencing non-smokers, particularly in the young, to become smokers of tobacco, one of the concerns of the WHO report (2016) was voiced by more than one respondent.

The clear lack of trust in commercial information sources and an associated lack of confidence in the knowledge of health professionals was evident. The distrust of current sources of information highlighted in this study has been reported previously (Dockrell *et al*., 2013; Hiscock *et al*., 2014) and corroborates evidence from studies of health professionals emphasising their lack of knowledge and leading to a reluctance on the part of smokers to approach their GP or practice nurse for advice (Gorzkowski *et al*., 2016; Marques Gomes *et al*., 2016; Van Gucht and Baeyens, 2016).

These smokers seem to regard e-cigarettes as a commercial product rather than as a medication, such as NRT products, for smoking cessation. Their awareness of the lack of strong evidence for the effectiveness and safety of e-cigarettes was apparent, leading to apprehension and hesitation when considering the possibility of e-cigarettes being made available on prescription by the NHS. An interesting observation in several participants was the comparison with the introduction of manufactured cigarettes in the early 20th century, and the time lag between their introduction and the discovery of the link with disease. This suggests that smokers, despite their reluctance to try to quit, are very aware of the risks of their behaviour, but tend to have an attitude of ‘stick with the devil I know’ rather than to try something that is new and the risks as yet unknown. The recurring appeal for further research and objective information make it clear that more reliable and accurate information regarding e-cigarettes is urgently needed, allowing smokers to make an informed choice of e-cigarettes as a smoking cessation method, and to aid their choice within the vast range.

Since this study was conducted the Tobacco Products Directive has been implemented, introducing mandatory safety and quality requirements for nicotine containing e-cigarettes for sale in the European Union. This is a forward step and the continuation of the regulation of e-cigarettes could lead to less uncertainty and misunderstanding in their use. However, only when sufficient evidence has demonstrated their health benefits and effectiveness would these smokers be reassured and accept prescriptions for e-cigarettes from the NHS.

### Strengths and weaknesses

Our findings are largely consistent with much of the existing literature surrounding the perception of e-cigarettes. However, most previous research has focussed on the perceptions and attitudes of current e-cigarette users, those of smokers attempting to quit and of ex-smokers. Limited research has been carried out amongst current tobacco smokers, and this is one of the few studies to use a qualitative approach to explore the views of this population. Semi-structured interviews allowed participants in this study to express themselves freely and enabled rich data collection. Participants represented a wide age range, and an almost equal male to female ratio allowing great diversity of views. None of the participants were currently using e-cigarettes, although some had previously tried. Nonetheless views tended to remain the same within both groups with no noticeable difference. However, due to the patient demographic in the GP practice, most participants were Caucasian, thus, racial and ethnic differences amongst perception and knowledge of e-cigarettes were not highlighted (Webb Hooper and Kolar, 2017) and the results are not generalisable to the UK population. Participants were recruited from a practice where the researcher may have been known to them, thus may have been more inclined to give favourable answers, or withhold views. Due to fear of judgement and opinions affecting their care at the practice. However, it was reiterated at various stages that all information would be confidential and not impact on their care. All of the interviews were carried out by one researcher, as was the main analysis, which, while allowing for greater familiarisation of the data introduced the potential for subjectivity and researcher bias, influencing the interpretation of the data. Although we endeavoured to minimise this through review and discussion, future research would adopt further measures to reduce any bias.

Also due to the time constraints only one method of qualitative data collection was used. The use of other methods such as focus groups would have enabled triangulation to take place further validating the study.

Nevertheless, these findings are relevant and can help to address the barriers to the use of e-cigarettes as an aid to smoking cessation in order to promote wider use for this purpose.

### Implications and further research

The findings of this study can have important implications for future education and research. The inconsistent knowledge amongst smokers surrounding e-cigarettes and the uncertainty and lack of confidence in the knowledge of health professionals calls for rapid action to implement more education in this sector, particularly in primary care. Providing healthcare professionals with greater knowledge of e-cigarettes would encourage smokers to seek unbiased sources of information to clarify the concerns highlighted in this research. The views of these participants also highlight the concerns of the general public of the long-term effects of e-cigarette use and of their effectiveness as an aid to smoking cessation. Further research into such areas is urgently required and will allow for greater understanding amongst smokers and healthcare professionals of the use of e-cigarettes as a long-term replacement for tobacco smoking. In addition, in view of the ever-changing demographics of the smoker population, qualitative research with specific age groups will be particularly useful, in particular to explore the concerns of e-cigarette use amongst younger and newer smokers.

## Conclusion

Overall smokers had a general awareness of e-cigarettes. While some viewed them to be healthier alternatives to smoking tobacco, concerns regarding their safety and efficacy as an aid to smoking cessation were common. The predominant theme of our study was that knowledge of the components of e-cigarettes is inconsistent and smokers are aware of the lack of strong evidence regarding health and efficacy as a smoking cessation aid. This acts as a barrier to their use as an aid to quitting, and further high-quality research is needed to alleviate their fears, if the opportunity to reduce tobacco consumption through the use of e-cigarettes is not to be lost.
